# Is STA really a low‐flow graft? A quantitative ultrasonographic study of the flow of STA for cerebral revascularization in MMD patients

**DOI:** 10.1111/cns.14197

**Published:** 2023-04-01

**Authors:** Yunyu Wen, Yanxia Gou, Baoping Wang, Zhibin Wang, Siyuan Chen, Shichao Zhang, Guozhong Zhang, Mingzhou Li, Wenfeng Feng, Songtao Qi, Gang Wang

**Affiliations:** ^1^ Department of Neurosurgery, Nanfang Hospital Southern Medical University Guangzhou Guangdong China; ^2^ Laboratory for Precision Neurosurgery, Nanfang Hospital Southern Medical University Guangzhou Guangdong China; ^3^ Department of Stomatology, Nanfang Hospital Southern Medical University Guangzhou Guangdong China; ^4^ Department of Ultrasound, Nanfang Hospital Southern Medical University Guangzhou Guangdong China

**Keywords:** cerebrovascular disease, Moyamoya disease, quantitative ultrasonography, STA‐MCA bypass, stroke

## Abstract

**Objective:**

Direct revascularization remains an important tool in the treatment of patients with Moyamoya disease (MMD). The superficial temporal artery (STA) is the most commonly used donor vessel for direct bypass, and an STA graft has traditionally been considered a low‐flow graft for flow augmentation. This study aimed to quantitatively evaluate the blood flow of the STA after direct revascularization.

**Methods:**

All direct revascularization procedures performed between 2018 and 2021 by one experienced neurosurgeon were screened. Quantitative ultrasound was used to measure the flow data of the patient's bilateral parietal branch of the STA(STA‐PB), the bilateral frontal branch of the STA(STA‐FB), and the left radial artery. Data on the patients' basic information, Suzuki grade, Matsushima type, anastomosis type, and blood biochemical parameters were collected and analyzed using univariate and multivariate models. An MBC Scale scoring system was proposed to evaluate the recipient artery network of the middle cerebral artery (MCA) tree. The relationship between MBC Scale score and STA graft flow was statistically analyzed.

**Results:**

In total, 81 patients (43 males and 38 females) successfully underwent STA‐MCA bypass and were included in this study. The mean flow rates in the STA‐PB graft on 1 day preoperatively, 1 day postoperatively, 7 days postoperatively, and >6 months postoperatively (long‐term) were 10.81, 116.74, 118.44, and 56.20 mL/min respectively. Intraoperative graft patency was confirmed in all patients. Comparing the preoperative and all postoperative time points, the STA‐PB flow rates were statistically significant (*p* < 0.001). The MCA‐C score was significantly associated with postoperative flow rate on day 1 (*p* = 0.007).

**Conclusion:**

The STA is a useful donor artery for direct revascularization inpatients with MMD and can provide sufficient blood supply to the ischemic cerebral territory.

## Graphical abstract

We conducted a single‐center retrospective study of MMD patients who received STA‐PB bypass surgery in our center from January 2018 to January 2021. The aim of this study was to quantitatively evaluate the blood flow of the STA‐PB after direct revascularization.

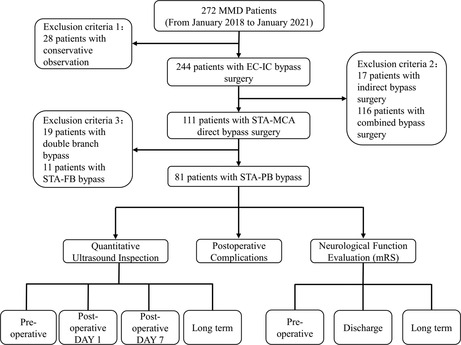

## 1 INTRODUCTION

Moyamoya disease (MMD) is a unique chronic progressive cerebrovascular disease characterized by stenosis or occlusion of the bilateral arteries around the circle of Willis, accompanied by the formation of arterial collateral circulation.^1^, ^2^ The prevalence of MMD range from 0.35 to 0.94 per 100,000 populations, and its sex ratio (women to men) is 1.8.^3^, ^4^, ^5^ The pathogenesis of MMD remains not clear. RNF213 is a susceptibility gene for MMD.^6^, ^7^ Patients with MMD show different clinical symptoms, such as a transient ischemic attack, ischemic and hemorrhagic stroke, and epilepsy.^8^, ^9^

Currently, a cure for MMD is not yet available.^2^ Improvement in cerebral blood flow (CBF) may protect against stroke in the future. Strategies for the surgical treatment of patients with MMD remain controversial. Several experienced surgeons recommend a combination of direct and indirect approaches.^10^ For adult patients with MMD, direct bypass is recommended as the first‐line treatment strategy. The superficial temporal artery‐middle cerebral artery (STA‐MCA) bypass is the most commonly used procedure to restore blood flow (BF) in the ischemic brain. Traditionally, the STA was considered a low‐flow bypass because the flow provided by the STA was thought to be approximately 10–20 mL/min. However, the STA can provide a more robust flow as a donor vessel. In Charbel's study, the cut flow of the STA ranged from 8 to 186 (median, 68) mL/min, and the bypass flows ranged from 1 to 154 (median, 48) mL/min.^11^ In another clinical report with a small sample size, the postoperative STA graft flow measured using quantitative magnetic resonance angiography (MRA) showed a mean value of 79 mL/min.^12^ However, the factors affecting STA graft flow remain unknown.

Several imaging examinations, including digital subtraction angiography (DSA), computed tomography angiography (CTA), and MRA, are used to detect whether a successful bypass treatment.^13^, ^14^, ^15^ DSA remains the gold standard for its detection.^16^ However, DSA is an invasive and costly technique. CTA and MRA can also be used for posttreatment evaluation.^17^, ^18^ However, CTA and MRA cannot detect CBF, flow rate, or blood supply range. Additionally, these two modalities are relatively costly.

Quantitative ultrasonography, a noninvasive inspection and cost‐effective method, can serve as an alternative tool to assess bypass patency.^19^ Blood vessel diameter, b BF velocity (BFV), and pulsation index (PI) score can be determined using quantitative ultrasonography. Moreover, it can detect changes at any time point during the perioperative period. The results of direct revascularization can be easily assessed using quantitative ultrasonography.

In this study, we aimed to detect the BF of the blood supply artery after direct revascularization and analyze the factors that affect BF.

## 2 MATERIALS AND METHODS

### 2.1 Patient selection

**FIGURE 1:**
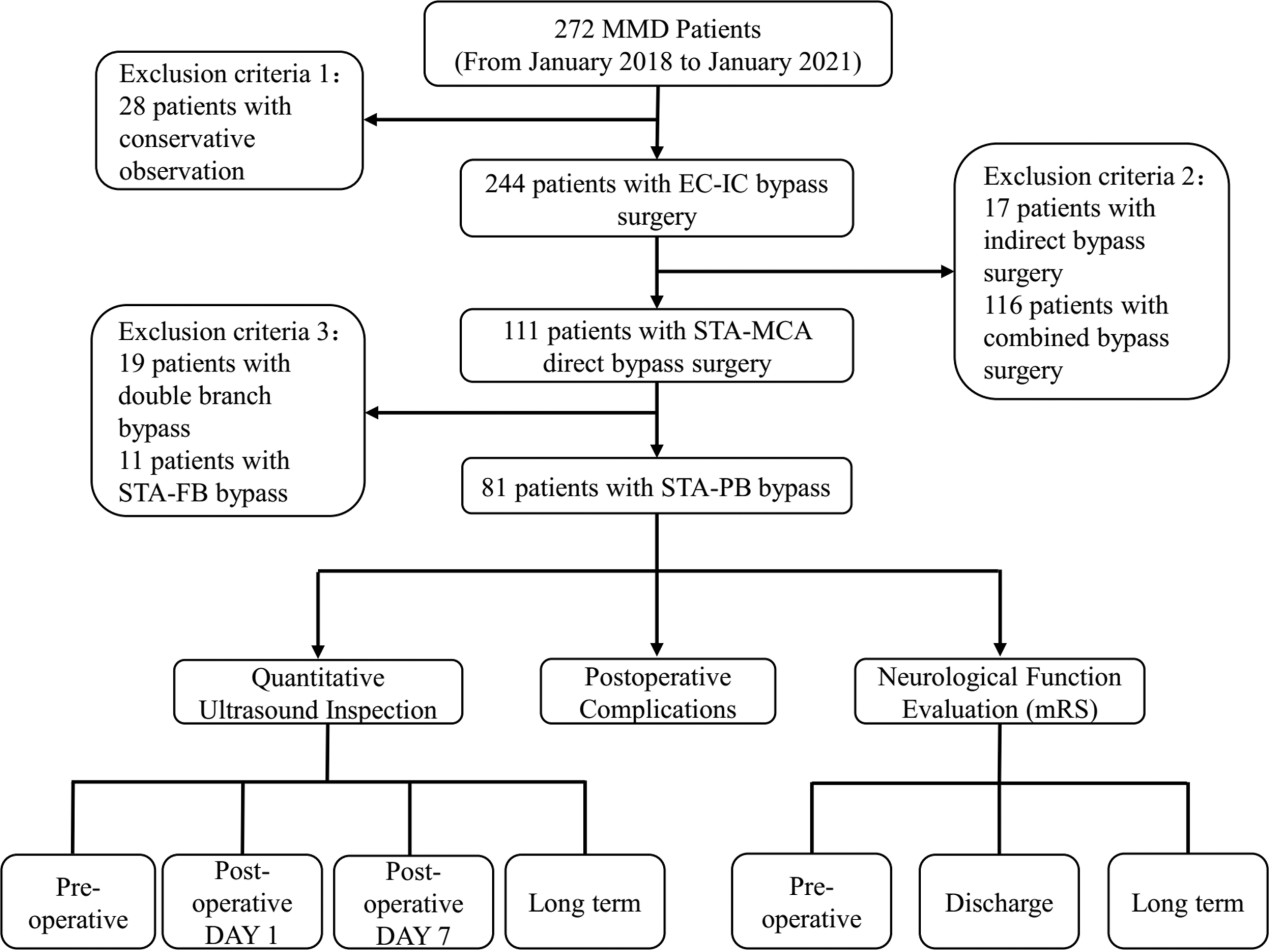
Flow diagram of the study population.

Patients with MMD who underwent STA‐MCA bypass surgery at Nanfang Hospital between January 2018 and January 2021 were enrolled in this study (Figure 1). Quantitative ultrasonography was performed within 24 h before DSA in all patients with MMD. All the surgeries were performed by an experienced clinical neurosurgeon. This study was approved by the Institutional Review Board of Nanfang Hospital (NFEC‐2022‐442). All patients provided informed consent before inclusion in this study.

### 2.2 Brief description of surgical procedure

All bypasses were performed by end‐to‐side anastomosis of the parietal branch of the STA (STA‐PB) to the MCA (frontal and/or temporal M4 branches of the MCA). In brief, the 10‐CM STA‐PB was dissected. Craniotomy was then performed. After opening the dura, one branch of the MCA was selected as the recipient vessel and trapped using two temporary clips placed 1 cm apart. A longitudinal incision was made in the recipient vessel, which was irrigated with heparin solution. The stump of the donor vessel was shaved obliquely to fit the recipient's side. A total of 8–12 interrupted sutures were made, with 3–5 on each side. Indocyanine green video angiography (performed using a commercially available microscope, OPMI Pentero, Carl Zeiss Co, Oberkochen, Germany) was used to validate the patency of the anastomosis.

### 2.3 Quantitative ultrasonography

Quantitative ultrasonography images were blindly and independently reviewed by two senior sonologists. The measurement method and precautions for quantitative ultrasonography were performed, as described previously.^19^ In brief, the patients were examined in the supine position. The checkpoint was located in the segment, where the STA passed through the skull bone. The diameter(D), flow, PI score, resistance index (RI) score, and mean flow velocity (Vm) of the STA‐PB, frontal branch of the STA (STA‐FB), and radial artery (RA) were recorded in the same patient at 1 day preoperatively (pre), 1 day postoperatively (day1), 7 days postoperatively (day7) and >6 months postoperatively (long term). The DSA images were independently analyzed by two senior neuroradiologists.

### 2.4 MBC Scale

**FIGURE 2:**
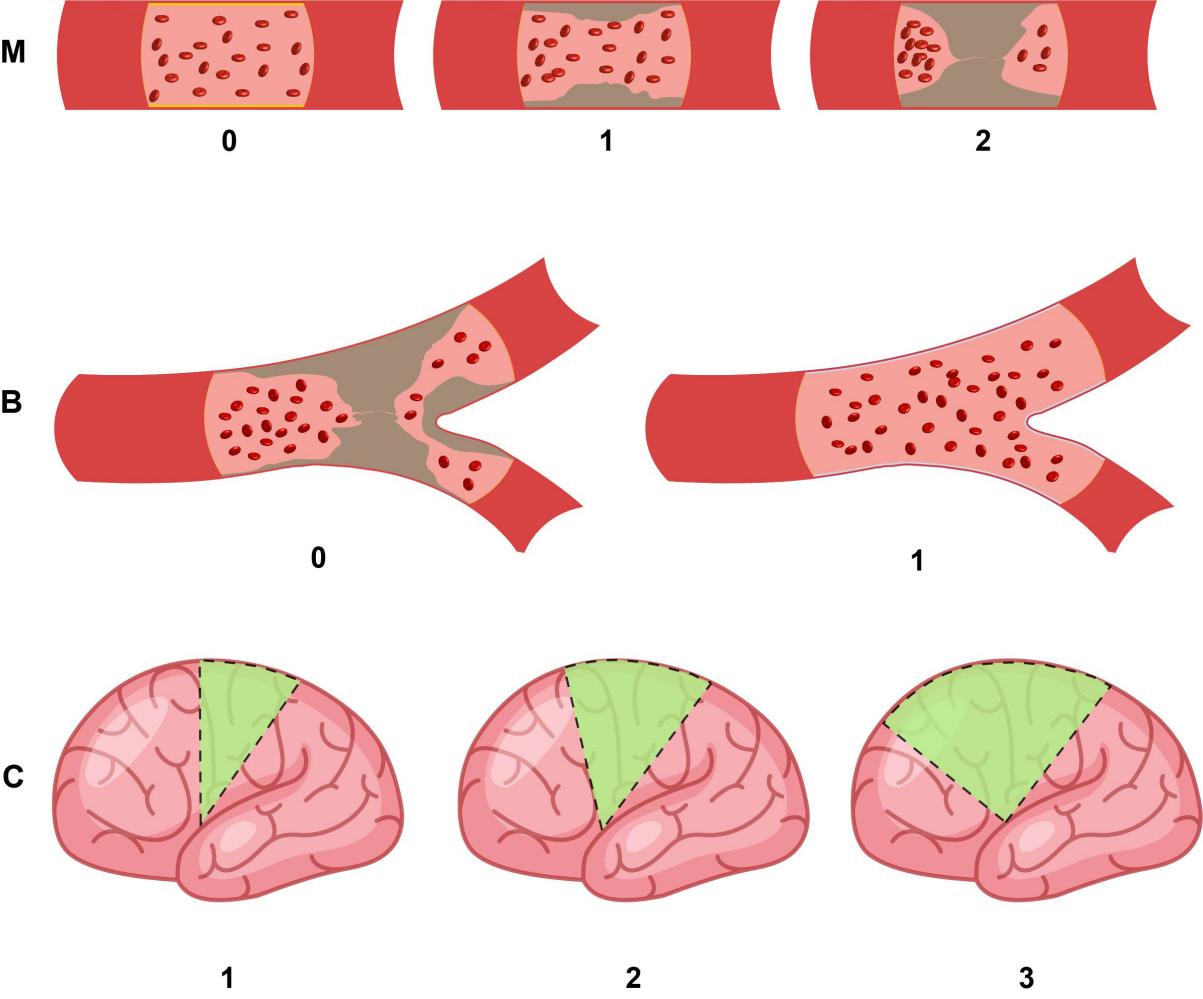
MBC Scale. M (M1 lumen: 0, normal; 1, stenosis; 2, occlusion). B (MCA bifurcation: 0, MCA bifurcation occlusion; 1, MCA bifurcation and communication between the blood vessels are normal). C (Cortical vascular integrity: 3, more than two‐thirds of the MCA cortical arterial branches can be visualized on preoperative digital subtraction angiography; 2, one‐third to approximately two‐thirds can be visualized; 1, less than one‐third can be visualized).

After the STA‐MCA bypass surgery, the flow of the STA graft was dependent on the integrity and capacity of the MCA network. Based on the DSA findings of patients with MMD, we proposed a new scale to assess the MCA network, mainly including the M1 lumen, MCA bifurcation, and cortical vascular integrity (Figure 2). The scale was scored as follows: (1) M1 lumen (M): 0, normal; 1, stenosis; 2, occlusion. (2) MCA bifurcation (B): 1, the MCA bifurcation and the communication between the blood vessels are normal; 0, MCA bifurcation occlusion. (3) cortical vascular integrity (C) is refer to the Matsushima grading:^20^ 3, more than two‐thirds of the MCA cortical arterial branches can be visualized on preoperative DSA (Grade A); 2, one‐third to approximately two‐thirds can be visualized (Grade B); 1, less than one‐third can be visualized (Grade C). The total score of M + B + C ranged from 1 to 6 points. A higher score indicated a better MCA network, indicating that more blood could be received from the donor artery.

### 2.5 Follow‐up

Patients who underwent STA‐MCA bypass surgery were followed up at least once in an outpatient clinic visit 6 months after the procedure and annually thereafter by telephone interviews or clinic visits. Quantitative ultrasonography and modified Rankin Scale (mRS) score assessment were performed during the outpatient follow‐up, with mRS scores being evaluated via telephone interviews. Quantitative ultrasonography was performed blindly and independently by two sonologists at each time point, and the mRS was followed up by two neurosurgeons in our research group.

### 2.6 Statistical analyses

Continuous variables are reported with mean ± standard deviation and were compared among groups using two‐way within‐subject analysis of variance (ANOVA) and Fisher's least significant difference test as post hoc comparisons. Comparisons between dependent results were performed using Student's paired t test. Categorical variables are presented as numbers and percentages. Changes in the quantitative ultrasonography indices over time were indicated using line charts and compared between different paired parameters, including STA‐PB/STA‐FB, STA‐PB/RA, and bypass/contralateral. Pearson's correlation coefficient analysis was performed to determine the relationship between continuous variables. Univariate and multivariate linear regression models were used to investigate the relationship between the independent variables and postoperative flow rates at day 1, day 7, and >6 months (long term). Significant independent variables in the univariate analysis were entered into the multivariate model. Significant independent variables in the multivariate model were recognized as variables associated with the outcome. All analyses were performed using the Statistical Package for the Social Sciences (SPSS) version 25 (SPSS Statistics V25, IBM Corporation, Somers, New York, USA). The statistical significance level for all tests was set at a two‐tailed *p* value of <0.05.

## 3 RESULTS

### 3.1 Participant's clinical characteristics

A total of 81 patients were included in this study. The average age of the patients was 43.42 ± 14.19 (range, 5–67 years), and the sex ratio was 1:0.88 (male/female, =43/38). In total, 42 (51.85%) and 39 (48.15%) patients underwent left and right bypasses, respectively. The characteristics of the 81 patients included in the analysis are shown in Table 1.

**TABLE 1:** The characteristics of 81 patients were included in regression analysis.

Parameters	*N* (%)	Mean ± SD	Median (range)
Sex			
Male	43 (53.09%)		
Female	38 (46.91%)		
Age, year		43.42 ± 14.19	48 (5 to 67)
Bypass side			
Left	42 (51.85%)		
Right	39 (48.15%)		
Disease history			
Hypertension	14 (17.28%)		
Diabetes	9 (11.11%)		
Hyperlipidemia	6 (7.41%)		
MCA		4.43 ± 0.91	4 (3 to 6)
M		1.71 ± 0.56	2 (0 to 2)
B		0.44 ± 0.50	0 (0 to 1)
C		2.28 ± 0.71	2 (1 to 4)
Suzuki grade (bypass side)		3.84 ± 1.65	4 (0 to 6)
Matsushima type		4.32 ± 1.78	5 (0 to 6)
Hb			
Preoperative		131.99 ± 16.95	132 (76 to 167)
Postoperative		118.95 ± 17.01	122 (71 to 160)
RBC			
Preoperative		4.52 ± 0.67	4.50 (1.05 to 6.57)
Postoperative		4.11 ± 0.44	4.16 (2.73 to 5.17)
PLT			
Preoperative		237.51 ± 78.60	222 (102 to 726)
Postoperative		221.58 ± 70.37	214 (108 to 540)
Intraoperative cortical artery stage		2.68 ± 0.67	3 (0 to 3)
Anastomosis type			
Straight incision	19 (23.46%)		
Oval shapes	47 (58.02%)		
Fish‐mouthing	15 (18.52%)		
Anastomosis needles		10.19 ± 2.18	10 (8 to 19)

Abbreviations: Hb, Hemoglobin; RBC, Red blood cell; PLT, Platelet.

### 3.2 The results of all ultrasonography indices

The results of all ultrasonography indices over time (Pre, day 1, day 7, >6 months [long term]), including D, flow, PI, RI, and Vm are shown in Table 2 and Table S1. The vessels included the STA‐PB and STA‐FB on the bypass side, the STA‐PB and STA‐FB on the contralateral side, and the RA.

**TABLE 2:** The results of all ultrasonography indices for the bypass side.

Parameters	Vessel	Mean ± SD			
		Preoperative	Post‐Day 1	Post‐Day 7	Long term
D bypass	PB	0.96 ± 0.26	1.83 ± 0.33	1.86 ± 0.37	1.60 ± 0.11
	FB	0.84 ± 0.18	1.24 ± 0.52	1.11 ± 0.22	0.94 ± 0.04
	RA	2.01 ± 0.35	2.17 ± 0.40	2.13 ± 0.58	2.01 ± 0.08
flow bypass	PB	10.81 ± 10.23	116.74 ± 71.42	118.44 ± 58.80	56.20 ± 15.19
	FB	6.62 ± 3.83	25.28 ± 46.55	16.91 ± 9.45	9.06 ± 1.46
	RA	34.38 ± 24.71	45.56 ± 31.01	38.56 ± 41.67	34.08 ± 3.18
PI bypass	PB	1.63 ± 0.47	0.87 ± 0.60	0.95 ± 1.35	0.90 ± 0.33
	FB	1.71 ± 0.48	1.68 ± 0.60	1.46 ± 0.51	1.45 ± 0.08
	RA	3.62 ± 1.89	3.68 ± 1.55	3.65 ± 1.64	3.16 ± 0.24
RI bypass	PB	0.74 ± 0.08	0.53 ± 0.14	0.50 ± 0.13	0.55 ± 0.04
	FB	0.76 ± 0.08	0.75 ± 0.09	0.70 ± 0.08	0.73 ± 0.02
	RA	0.88 ± 0.11	0.90 ± 0.09	0.89 ± 0.11	0.87 ± 0.02
Vm bypass	PB	21.45 ± 8.56	69.99 ± 36.76	71.46 ± 40.37	21.06 ± 3.56
	FB	19.08 ± 7.25	28.32 ± 8.73	27.78 ± 10.26	10.52 ± 1.01
	RA	16.76 ± 8.85	19.30 ± 8.95	16.77 ± 7.98	9.42 ± 0.75

### 3.3 Comparisons among time points

**FIGURE 3:**
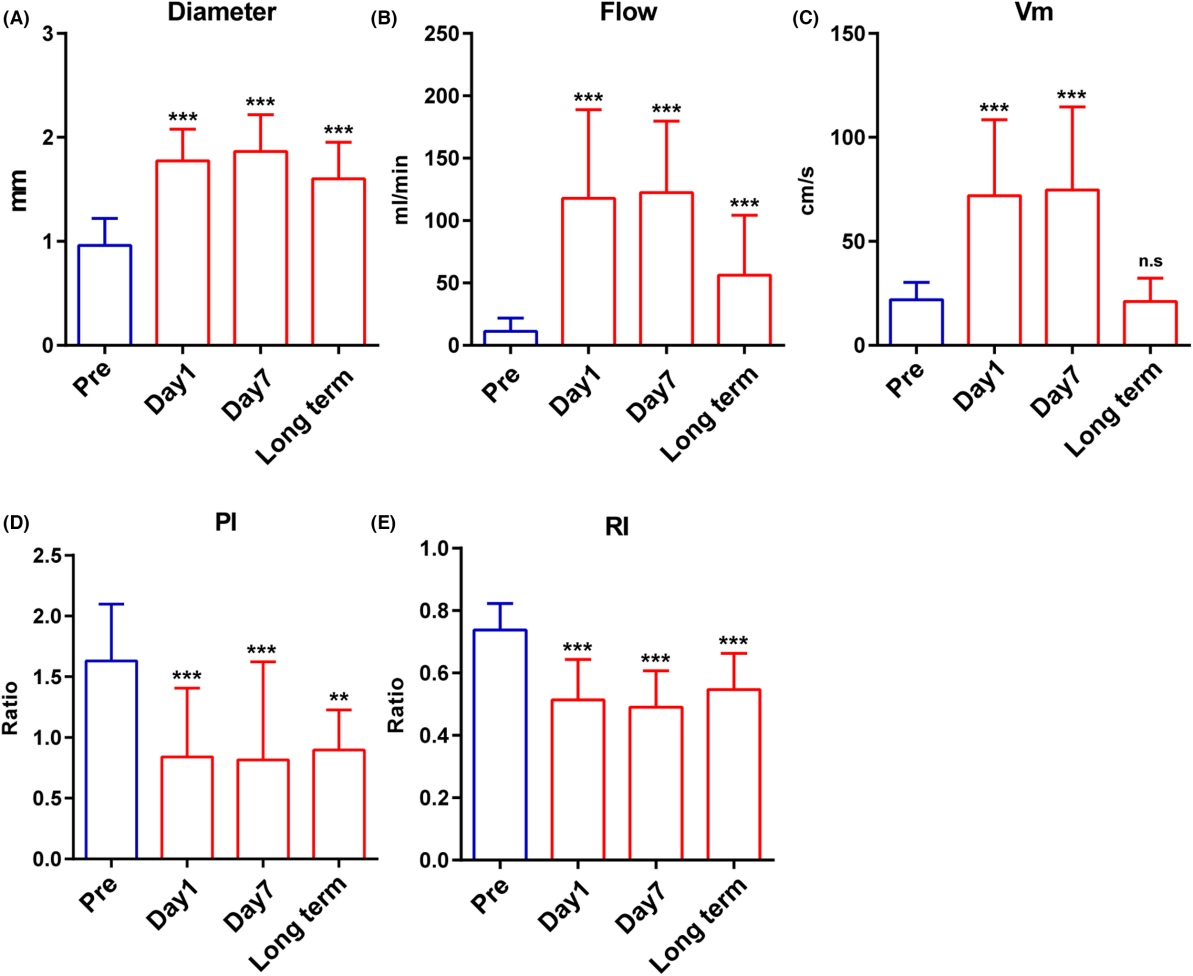
The ultrasonography indices of the STA‐PB at the bypass side in four time points, including diameter (A), flow (B), Vm (C), PI (D), and RI (E). All postoperative data are compared with preoperative data (**p < 0.01; *** *p* < 0.001; n.s, no significance).

Figure 3 and Table S2 further present the significance of the ultrasonography indices among the four time points. The most important observation was that the results of 1 day preoperatively versus 1 day postoperatively (pre vs day1), 1 day preoperatively versus 7 days postoperatively (pre vs day7), and 1 day preoperatively versus >6 months (long‐term) (pre vs long term) were significant (all *p* < 0.05). These results again confirmed the changes in the indices of the STA‐PB after bypass surgery, and the changes remained the same between days 1 and 7. The diameter and flow showed a significant decrease in the long‐term period but were still significantly higher than the preoperative values. The Vm decreased in the long‐term period to the same level as the preoperative value.

### 3.4 Comparisons of all indices between the parietal branch of the superficial temporal artery (STA‐PB) at the bypass side and radial artery

**FIGURE 4:**
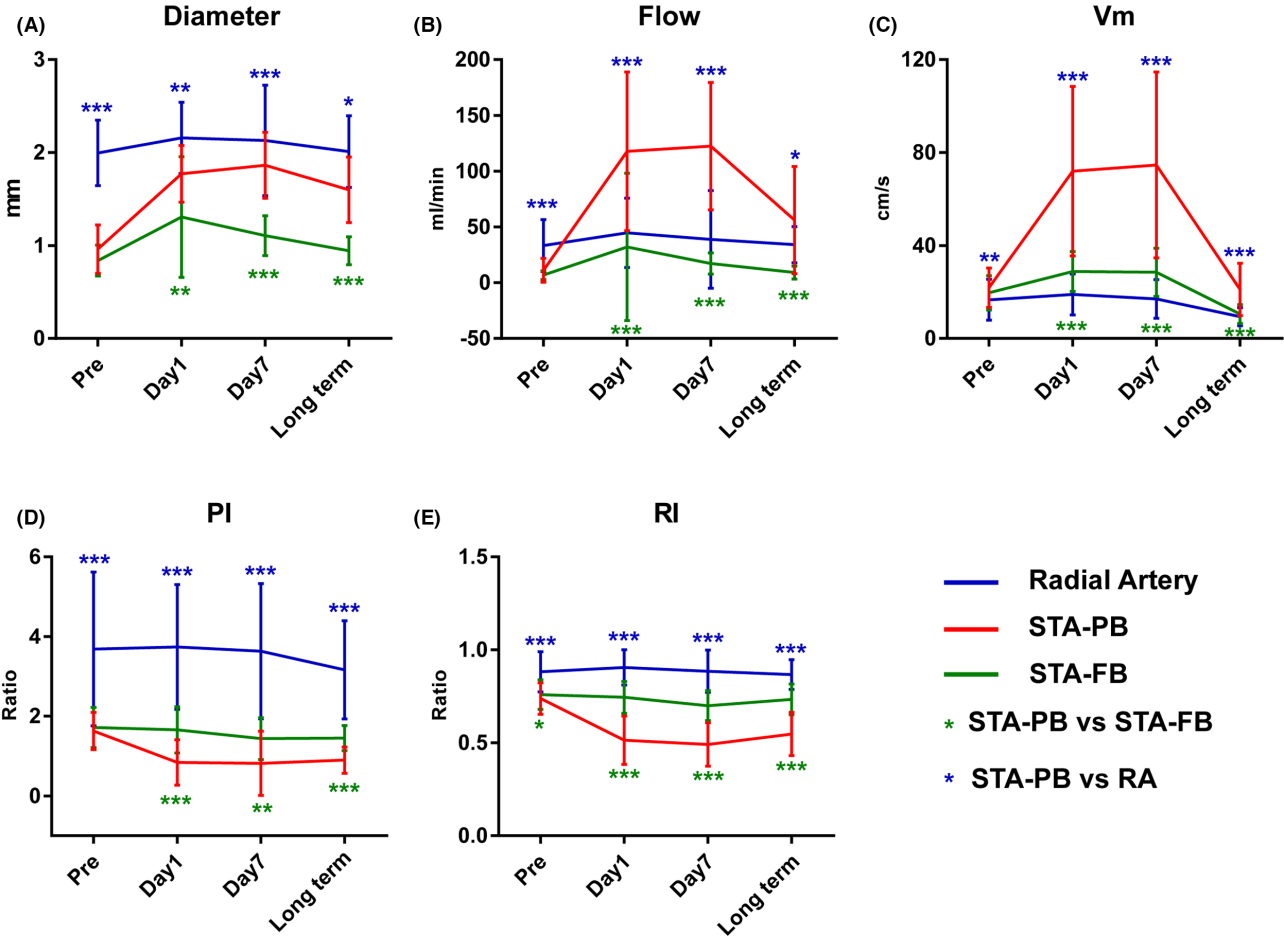
The ultrasonography indices of the STA‐PB at the bypass side, the STA‐FB at the bypass side, and the RA over time, including diameter (A), flow (B), Vm (C), PI (D), and RI (E). (STA‐PB, The parietal branch of the superficial temporal artery; STA‐FB, frontal branch of the superficial temporal artery; RA, radial artery; Vm, mean flow velocity; PI, pulsation index; RI, resistance index; *p < 0.05; **p < 0.01; *** *p* < 0.001).

In paired comparisons, all STA‐PB indices on the bypass side were significantly different from the corresponding RA indices postoperatively (all *p* < 0.05, Table 2). As shown in Figure 4, the flow was higher in the RA than in the STA‐PB before bypass surgery (*p* < 0.001); however, after bypass surgery, the flow was significantly higher in the STA‐PB than in the RA (all *p* < 0.05). The diameter, PI and RI were higher in the RA than in the STA‐PB at any time points (all *p* < 0.05), however, the Vm were lower in the RA than in the STA‐PB at any time points (all *p* < 0.01).

### 3.5 ANOVA results and comparison between the STA‐PB and STA‐FB at the bypass side

Figure 4 and Table S3 show the results of ANOVA and comparison between the STA‐PB and STA‐FB at the bypass side. In these analyses, we aimed to confirm whether the flow and indices would significantly change in the STA‐PB after bypassing using the STA‐FB as a control. In the *p* value results at the bypass side, the results confirmed that after bypass surgery, significantly lower PI, RI, and significantly higher D, flow, and Vm were observed in the STA‐PB than in the STA‐FB (all *p* < 0.05). The significance of the interaction effect was caused by a small difference (D, flow, RI) and insignificance (PI, Vm) between the STA‐PB and STA‐FB before surgery (means and P values are indicated in Table S1 and Table 2).

### 3.6 Comparisons of all indices between the bypass and contralateral sides

**FIGURE 5:**
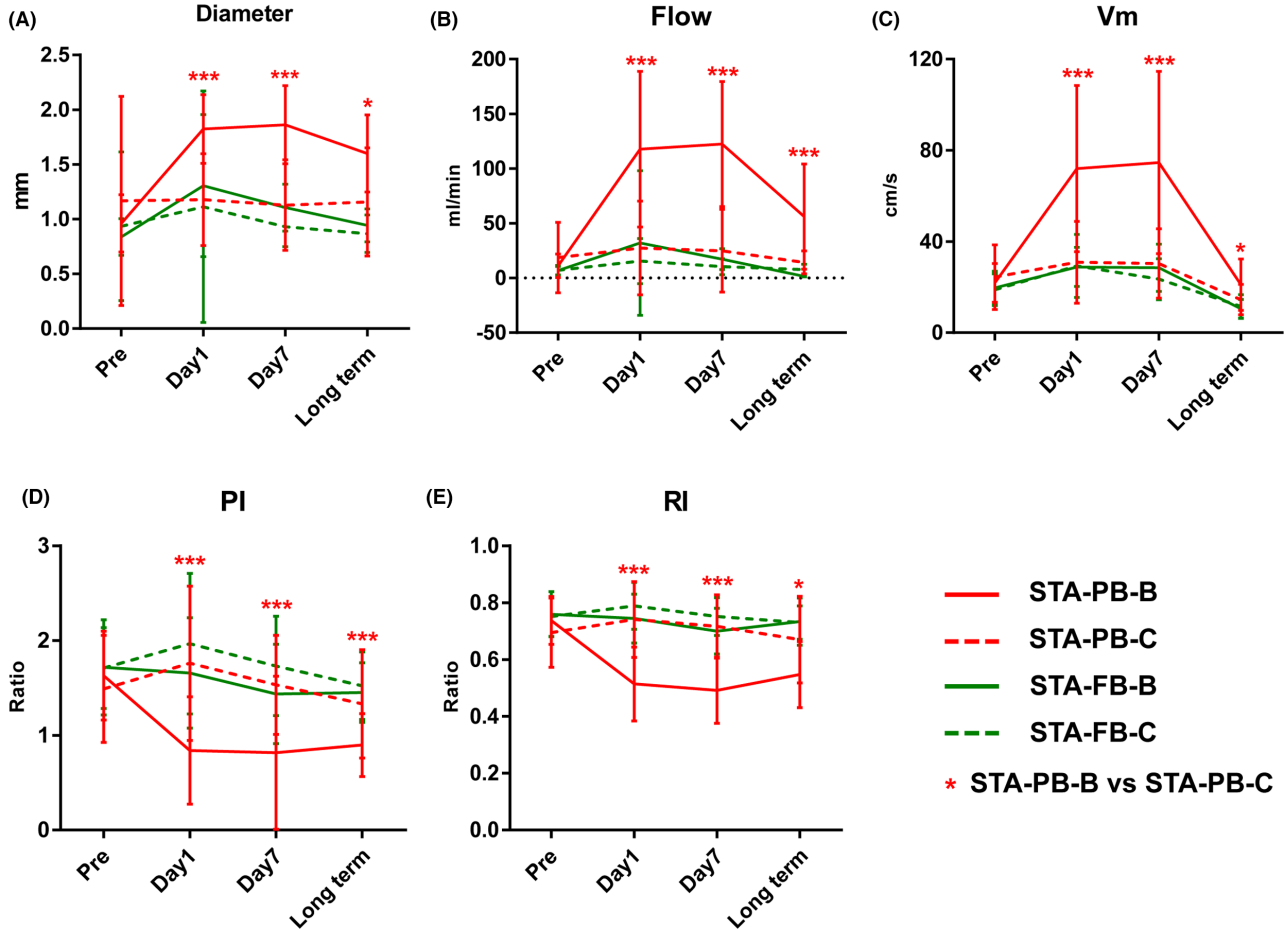
The ultrasonography indices of the STA‐PB and STA‐FB between the bypass and contralateral sides over time, including diameter (A), flow (B), Vm (C), PI (D), and RI (E) (STA‐PB‐B, bypass side of the parietal branch of the STA‐PB; STA‐PB‐C, contralateral side of the STA‐PB; STA‐FB‐B, bypass side of the STA‐FB; STA‐FB‐C, The contralateral side of the frontal branch of the STA‐FB; STA‐PB, parietal branch of the superficial temporal artery; STA‐FB, frontal branch of the superficial temporal artery; Vm, mean flow velocity; PI, pulsation index; RI, resistance index; *p < 0.05; *** *p* < 0.001).

The significance of all indices between the bypass and contralateral sides is shown in Table 2. Furthermore, line charts over time are shown, including the STA‐PB and STA‐FB, in Figure 5. The contralateral side could be considered an alternative control reference for bypass results. These results confirm this hypothesis. All ultrasonography indices in STA‐PB significantly differed between the bypass and contralateral sides (all *p* < 0.05). Particularly after surgery, the pattern was similar to the comparison between the STA‐PB and STA‐FB at the bypass side. Significant differences were observed in the STA‐FB between the bypass and contralateral sides; however, no specific pattern is shown in Figure 5.

### 3.7 Associated factors of postoperative flow on days 1 and 7

In the correlation coefficient analysis, no significance was found between all preoperative ultrasonography indices of the STA‐PB and the postoperative flow at day 1 and day 7 (r range from −0.08 to 0.18, all *p* > 0.05).

All patients in this study had comparatively complete records of clinical characteristics, including disease type, disease history, MCA score, Suzuki grade, Matsushima type, hemoglobin (Hb) level, red blood cell (RBC) level, platelet (PLT) level, intraoperative cortical artery stage, and anastomosis type and needles used (Table 1). The data from the 81 patients in this study were further used to investigate the relationship between the independent variables and postoperative flow at days 1 and 7. MCA‐C score was significantly associated with postoperative flow at day 1 (B = 28.16; 95% confidence interval [CI] = 7.77–48.56; *p* = 0.007), and the postoperative flow at day 1 significantly predicted the flow at day 7 (B = 0.47; 95% CI = 0.31–0.63; *p* < 0.001) (Table 3 and Table S4).

**TABLE 3:** The associations of independent variables to postoperative STA‐PB flow at day 1.

Parameters	Univariate		Multivariate	
	Estimated B (95% CI)	*p*	Estimated B (95% CI)	*p*
Sex				
Male				
Female	−17.43 (−48.25 to 13.40)	0.264		
Age, year	0.19 (−0.91 to 1.29)	0.737		
Bypass side				
Left				
Right	1.98 (−29.05 to 33.01)	0.899		
Disease history				
Hypertension	9.90 (−31.05 to 50.85)	0.632		
Diabetes	36.69 (−11.96 to 85.34)	0.137		
Hyperlipidemia	1.57 (−57.64 to 60.77)	0.958		
MCA	15.19 (−1.50 to 31.89)	0.074		
M	−16.61 (−44.30 to 11.09)	0.236		
B	10.52 (−20.49 to 41.53)	0.501		
C	29.88 (9.11 to 50.65)	0.005	28.16 (7.77 to 48.56)	0.007
Suzuki grade (bypass side)	−3.51 (−12.90 to 5.87)	0.458		
Matsushima type	−4.99 (−13.87 to 3.88)	0.266		
Hb				
Preoperative	0.94 (0.04 to 1.83)	0.041	0.46 (−0.48 to 1.40)	0.336
Postoperative	0.63 (−0.28 to 1.53)	0.171		
RBC				
Preoperative	25.56 (2.99 to 48.12)	0.027	19.61 (−3.97 to 43.18)	0.102
Postoperative	29.46 (−5.06 to 63.98)	0.093		
PLT				
Preoperative	0.06 (−0.13 to 0.26)	0.525		
Postoperative	0.12 (−0.10 to 0.34)	0.295		
Intraoperative cortical artery stage	15.48 (−7.63 to 38.59)	0.186		
Anastomosis type				
Straight incision				
Oval shapes	1.42 (−30.00 to 32.84)	0.929		
Fish‐mouthing	−17.90 (−57.61 to 21.82)	0.372		
Anastomosis needles	−3.32 (−10.60 to 3.96)	0.366		

### 3.8 Postoperative complications

Among the 81 patients, the overall incidence of postoperative complications was approximately 11.1% (nine patients), including seizure, subcutaneous hematoma, transient aphasia, and pulmonary infection in three, two, two, and two patients, respectively.

### 3.9 Clinical outcome

In our study, all patients were followed up via telephone interviews or clinic visits. In total, 78 (96.3%) patients had mRS scores ranging from 0 to 2, and three patients had mRS scores >3(Table 4). One patient had mRS scores of 4 at discharge and 5 at 2 years after surgery.

**TABLE 4:** Patients' mRS scores at preoperative, discharge, and long‐term follow‐up.

	Preoperative	Postoperative	
		Discharge	Long term
mRS			
0–2	71 (87.7%)	75 (92.6%)	78 (96.3%)
3	8 (9.9%)	5 (6.2%)	2 (2.5%)
4	2 (2.5%)	1 (1.2%)	0 (0.0%)
5	0 (0.0%)	0 (0.0%)	1 (1.2%)

## 4 DISCUSSION

In our study, we performed quantitative ultrasonography in patients with MMD who underwent STA‐MCA bypass surgery 1 day preoperatively, 1 day postoperatively, 7 days postoperatively, and >6 months postoperatively. The diameter, flow, and Vm were significantly increased after bypass surgery. Diameter, flow, and Vm were significantly higher in the STA‐PB than in the STA‐FB. The flow of STA‐PB increased to 116.74 mL/min and 118.44 mL/min at 1 and 7 days after bypass surgery, respectively, and remained 56.2 mL/min >6 months after surgery. Although the Vm was not significantly different in the long‐term from the preoperative value, however, the vessel diameter remained high, which explains why the flow remained higher in the long‐term period. As a donor blood vessel, the RA is commonly used for bypass surgery in patients with coronary heart disease.^21^, ^22^ The RA is an ideal graft for bypass surgery in patients with MMD.^23^, ^24^ However, our study demonstrated that the STA‐PB could provide high flow. The STA‐PB is not traditionally conceived as a high‐flow graft, but our data objectively demonstrate its potential to provide higher flow in STA‐MCA bypass surgery. Compared with the RA, the STA as a graft for bypass surgery has the advantages of convenient operation and less trauma.^25^, ^26^, ^27^ When the RA is used as a graft for extracranial‐intracranial (EC‐IC) bypass surgery, two anastomoses need to be used, which increases the risk of occlusion. Therefore, the STA can be used as a donor artery for EC‐IC bypass surgery and can provide adequate blood supply to the brain. According to our MCA recipient network grading scale, the flow of graft is extremely related to the cortical artery of the MCA (*p* = 0.007), which means that the flow of the graft depends on the distribution of arteries in the cerebral cortex and is closely related to the blood requirement of the brain and the integrity of the cortical artery network. Moreover, the morphology of the anastomosis was not relevant to the STA flow (Table 3 and Table S4). However, the diameter of the anastomosis could not be measured.

The STA as a donor vessel for EC‐IC bypass surgery has been widely used in the treatment of cerebral ischemic diseases, such as MMD, intracranial aneurysms, occlusive cerebrovascular disease, and cranial base tumors.^17^, ^28^, ^29^, ^30^, ^31^ STA‐MCA bypass surgery is the preferred surgical method for treating MMD. Patients with complex cerebral aneurysms unsuitable for direct surgical clipping or endovascular embolization can also undergo STA‐MCA bypass surgery in combination with endovascular exclusion. The use of this method, called hybrid surgery, to treat complex or giant intracranial aneurysms has also been accepted by neurosurgeons increasingly.^32^, ^33^, ^34^ Our previous study revealed that STA grafts showed good patency after STA‐MCA bypass surgery in patients with intracranial aneurysms and that the mean flow could be increased above 100 mL/min with these grafts.^35^

We collected data from the STA‐PB, STA‐FB, and RA in the same patient at different time periods. Both RA and STA‐FB were used as negative controls. The STA‐PB had a significantly higher blood supply after surgery, which means that the BF to the trunk of the STA would also be significantly increased. In contrast to the STA‐FB, the downstream of the STA‐PB was changed, whereas the STA‐FB still supplied the scalp. No change was observed in the STA‐FB flow postoperatively, which remained at 10–20 mL/min. Thus, there was a significant increase in the STA‐PB, whereas the STA‐FB BF did not change, which is consistent with our proposed MBC Scale result that the greater the vascular network demand in the blood supply area, the more abundant the blood supply. The comparison with the RA was made, first, to determine whether the patients' cardiac function and blood pressure at that time would have an effect on the data collection to exclude errors arising from the measurement and, second, to demonstrate that although the diameter and BF of the STA‐PB were significantly lower than those of the RA preoperatively, the diameter of the STA‐PB had reached the level of the RA, and the flow significantly increased postoperatively; therefore, it was also feasible to use the STA‐PB as a graft.

Previous studies have used ultrasound to detect the STA‐MCA bypass flow. Kim et al. considered that when the mean flow rate increased by >47.5% and the cross‐sectional diameter increased by >15%, the patency of the anastomosis on DSA was good.^36^ Hirai et al. demonstrated that duplex ultrasonography was available for evaluating the postsurgical patency of bypass flow and regional cerebral blood flow (rCBF) in the ipsilateral MCA territory.^37^ In this study, we included patients who received only the STA‐PB as the donor artery and did not receive indirect bypass. Meanwhile, the indices used in this study were more complete, including flow, diameter, PI, RI, and mean velocity, whereas most previous studies have used data on flow rate, which cannot exclude velocity variations caused by stenosis. In addition, our study investigated the patency of the donor vessels and further explored the actual flow of the STA and related influencing factors.

Our study has some limitations. First, the number of patients enrolled in our study was small, particularly those with aneurysm. Increasing the proportion of patients in this study would be beneficial for our study. Second, to reduce human measurement and machine errors, patients could only return to our center for quantitative ultrasonography testing, resulting in a lack of long‐term data for most patients. Finally, we did not monitor the flow during surgery. If the flow can be detected intraoperatively, we can adjust the size of the anastomosis according to the intraoperative flow and analyze the effect of the intraoperative flow size on the short‐ and long‐term postoperative periods.

## 5 CONCLUSION

The STA can be used as a donor artery for EC‐IC bypass surgery and can provide a robust blood supply to the brain. The mean STA flow rate can reach 116.74 mL/min and 118.44 mL/min 1 day and 1 week postoperatively, respectively, and can also be maintained at 56.2 mL/min for >6 months after surgery.

## AUTHOR CONTRIBUTIONS

Gang Wang and Wenfeng Feng designed the study. Yunyu Wen and Yanxia Gou analyzed the data. Baoping Wang performed quantitative ultrasound examinations on all patients. Yunyu Wen and Gang Wang drafted the manuscript. Siyuan Chen, Shichao Zhang, Guozhong Zhang, and Mingzhou Li collected the data. Wenfeng Feng and Songtao Qi critically revised the manuscript. All the authors contributed to the manuscript article and approved the submitted version.

## FUNDING INFORMATION

This work was funded by grants from the Guangdong Basic and Applied Basic Research Foundation (No. 2020A1515110695), the Science and Technology project of Maoming (No. 2021S0049), and the President Foundation of Nanfang Hospital, Southern Medical University (No.2020C035).

## CONFLICT OF INTEREST STATEMENT

The authors declare that this study was conducted in the absence of any commercial or financial relationships that could be construed as a potential conflict of interest.

## ETHICS APPROVAL AND CONSENT TO PARTICIPATE

All procedures involving human participants were performed in accordance with the ethical standards of the Review Committee of the Nanfang Hospital Ethics Committee of Southern Medical University (NFEC‐2022‐442) and with the 1964 Declaration of Helsinki. Informed consent was obtained from all the participants.

## Supporting information


Table S1

## Data Availability

Data sharing not applicable to this article as no datasets were generated or analysed during the current study.
