# Supplementary material for: Is STA really a low‐flow graft? A quantitative ultrasonographic study of the flow of STA for cerebral revascularization in MMD patients
Source: CNS Neurosci Ther. 2023 Apr 1;29(9):2572–82. doi: 10.1111/cns.14197 (PMC10401118; doi:10.1111/cns.14197)
Supplement: Supplementary file 1 — Table S1 [file CNS-29-2572-s001.docx]

**Supplementary Table 1 The results of all ultrasonography indices for contralateral side**

|  |  | Mean ± SD | | | |
| --- | --- | --- | --- | --- | --- |
| Parameters | Vessel | Pre-operative | Post-Day1 | Post-Day7 | Long-term |
| D-contralateral | PB | 1.13±0.90 | 1.17±0.40 | 1.13±0.41 | 1.16±0.11 |
|  | FB | 0.92±0.63 | 1.10±0.99 | 0.94±0.18 | 0.87±0.04 |
| flow-contralateral | PB | 17.61±30.40 | 26.67±40.98 | 24.39±36.38 | 14.33±2.28 |
|  | FB | 6.99±4.39 | 15.97±19.65 | 10.61±6.96 | 7.78±1.13 |
| PI-contralateral | PB | 1.51±0.55 | 1.75±0.77 | 1.55±0.52 | 1.33±0.13 |
|  | FB | 1.70±0.43 | 1.93±0.73 | 1.73±0.50 | 1.52±0.08 |
| RI-contralateral | PB | 0.70±0.12 | 0.74±0.13 | 0.72±0.11 | 0.67±0.03 |
|  | FB | 0.75±0.07 | 0.78±0.08 | 0.75±0.07 | 0.73±0.01 |
| Vm-contralateral | PB | 24.22±13.80 | 30.62±17.66 | 29.81±14.73 | 14.51±1.46 |
|  | FB | 18.98±6.76 | 30.00±14.08 | 23.64±8.52 | 11.48±1.24 |

**Supplementary Table 2 The P-value of paired comparisons** **of STA-PB among time-points in ultrasonography indices**

|  | comparisons among time-points | | | |
| --- | --- | --- | --- | --- |
| Parameters | Pre vs Day1 | Pre vs Day7 | Pre vs Long term |  |
| D | <0.001 | <0.001 | <0.001 |  |
| flow | <0.001 | <0.001 | <0.001 |  |
| PI | <0.001 | <0.001 | 0.002 |  |
| RI | <0.001 | <0.001 | <0.001 |  |
| Vm | <0.001 | <0.001 | 0.805 |  |

**Supplementary Table 3 The ultrasonography indices comparisons between STA-PB and STA-FB in bypass side**

|  |  |  |  | P-value within time-point | | | |
| --- | --- | --- | --- | --- | --- | --- | --- |
|  | P-value of ANOVA effects | | | Pre | Day1 | Day7 | Long-term |
| Parameters | PB/FB | Time | Interaction | PB-FB | PB-FB | PB-FB | PB-FB |
| D | <0.001 | <0.001 | <0.001 | <0.001 | <0.001 | <0.001 | <0.001 |
| flow | <0.001 | <0.001 | <0.001 | 0.002 | <0.001 | <0.001 | <0.001 |
| PI | <0.001 | <0.001 | 0.003 | 0.104 | <0.001 | 0.017 | 0.601 |
| RI | <0.001 | <0.001 | <0.001 | 0.042 | <0.001 | <0.001 | <0.001 |
| Vm | <0.001 | <0.001 | <0.001 | 0.187 | <0.001 | <0.001 | 0.002 |

**Supplementary Table 4 The associations of independent variables to postoperative STA-PB flow at day7**

|  | Univariate | |  | Multivariate | |
| --- | --- | --- | --- | --- | --- |
| Parameters | Estimated B (95% CI) | P |  | Estimated B (95% CI) | P |
| Sex |  |  |  |  |  |
| Male |  |  |  |  |  |
| Female | -30.34 (-55.86 to -4.82) | 0.020 |  | -17.22 (-42.11 to 7.66) | 0.172 |
| Age, year | 0.29 (-0.64 to 1.21) | 0.541 |  |  |  |
| Bypass side |  |  |  |  |  |
| Left |  |  |  |  |  |
| Right | 21.74 (-4.31 to 47.79) | 0.100 |  |  |  |
| Disease history |  |  |  |  |  |
| Hypertension | 8.18 (-26.67 to 43.03) | 0.641 |  |  |  |
| Diabetes | 37.68 (-4.21 to 79.56) | 0.077 |  |  |  |
| Hyperlipidemia | 12.14 (-40.74 to 65.03) | 0.649 |  |  |  |
| MCA | 13.23 (-0.61 to 27.08) | 0.061 |  |  |  |
| M | -6.66 (-31.27 to 17.94) | 0.591 |  |  |  |
| B | 9.72 (-16.35 to 35.78) | 0.460 |  |  |  |
| C | 20.52 (3.05 to 37.99) | 0.022 |  | 5.80 (-8.97 to 20.57) | 0.436 |
| Suzuki grade (bypass side) | -2.46 (-10.48 to 5.57) | 0.543 |  |  |  |
| Matsushima type | -3.90 (-11.50 to 3.70) | 0.310 |  |  |  |
| Hb |  |  |  |  |  |
| Preoperative | 0.88 (0.11 to 1.65) | 0.026 |  | -0.05 (-0.82 to 0.72) | 0.889 |
| Postoperative | 0.77 (0.00 to 1.55) | 0.051 |  |  |  |
| RBC |  |  |  |  |  |
| Preoperative | 18.62 (-1.02 to 38.26) | 0.063 |  |  |  |
| Postoperative | 31.11 (-1.24 to 63.46) | 0.059 |  |  |  |
| PLT |  |  |  |  |  |
| Preoperative | 0.10 (-0.06 to 0.26) | 0.229 |  |  |  |
| Postoperative | 0.17 (-0.01 to 0.36) | 0.065 |  |  |  |
| Intraoperative cortical artery stage | 11.92 (-8.13 to 31.97) | 0.240 |  |  |  |
| Anastomosis type |  |  |  |  |  |
| Straight incision |  |  |  |  |  |
| Oval-shapes | 7.94 (-18.96 to 34.85) | 0.558 |  |  |  |
| Fish-mouthing | -15.92 (-50.62 to 18.78) | 0.364 |  |  |  |
| Anastomosis needles | 2.39 (-4.11 to 8.89) | 0.466 |  |  |  |
| STA flow (post-day1) | 0.52 (0.38 to 0.67) | <0.001 |  | 0.47 (0.31 to 0.63) | <0.001 |

**Abbreviations**

| Moyamoya disease | MMD |
| --- | --- |
| Superficial temporal artery | STA |
| Parietal branch of the superficial temporal artery | STA-PB |
| Frontal branch of the superficial temporal artery | STA-FB |
| Radial artery | RA |
| Middle cerebral artery | MCA |
| Cerebral blood flow | CBF |
| Digital subtraction angiography | DSA |
| Computed tomography angiography | CTA |
| Magnetic resonance angiography | MRA |
| Blood flow velocity | BFV |
| Blood flow | BF |
| Diameter | D |
| Pulsation index | PI |
| Resistance index | RI |
| Mean flow velocity | Vm |
| modified Rankin Scale | mRS |
| Pre-operative | Pre |
| Post-operative day 1 | Day1 |
| Post-operative day 7 | Day7 |
| More than 6 months postoperatively | Long-term |
| Standard deviation | SD |
| Pre-operative versus post-operative day 1 | Pre vs Day1 |
| Pre-operative versus post-operative day 7 | Pre vs Day7 |
| Pre-operative versus post-operative long term | Pre vs Long term |
| Hemoglobin | Hb |
| Red blood cell | RBC |
| Platelet | PLT |
| Extracranial-intracranial | EC-IC |
|  |  |
